# Autologous bone grafts with MSCs or FGF-2 accelerate bone union in large bone defects

**DOI:** 10.1186/s13018-016-0442-6

**Published:** 2016-09-26

**Authors:** Hiroaki Murakami, Tomoyuki Nakasa, Masakazu Ishikawa, Nobuo Adachi, Mitsuo Ochi

**Affiliations:** Department of Orthopaedic Surgery, Integrated Health Sciences, Institute of Biomedical and Health Sciences, Hiroshima University, 1-2-3 Kasumi, Minami-ku, Hiroshima, 734-8551 Japan

**Keywords:** Large bone defects, Autologous bone grafts, Atrophic non-union model, Mesenchymal stromal cells (MSCs), Fibroblast growth factor-2 (FGF-2)

## Abstract

**Bacground:**

Although the contribution of fibroblast growth factor (FGF)-2 and mesenchymal stromal cells (MSCs) to bone formation is well known, few studies have investigated the combination of an autologous bone graft with FGF-2 or MSCs for large bone defects.

**Methods:**

We studied an atrophic non-union model with a large bone defect, created by resecting a 10-mm section from the center of each femoral shaft of 12-week-old Sprague-Dawley rats. The periosteum of the proximal and distal ends of the femur was cauterized circumferentially, and excised portions were used in the contralateral femur as autologous bone grafts. The rats were randomized to three groups and given no further treatment (group A), administered FGF-2 at 20 μg/20 μL (group B), or 1.0 × 10^6^ MSCs (group C). Radiographs were taken every 2 weeks up to 12 weeks, with CT performed at 12 weeks. Harvested femurs were stained with toluidine blue and evaluated using radiographic and histology scores.

**Results:**

Radiographic and histological evaluation showed that bone union had been achieved at 12 weeks in group C, while group B showed callus formation and bridging callus but non-union, and in group A, callus formation alone was evident. Both radiographic and histological scores were significantly higher at 2, 4, 6, 8, 10, and 12 weeks in groups B and C than group A and also significantly higher in group C than group B at 12 weeks.

**Conclusions:**

These data suggest that autologous bone grafts in combination with MSCs benefit difficult cases which cannot be treated with autologous bone grafts alone.

## Background

For bone defects or non-union, autologous bone grafts are used daily in clinical practice. Although autologous bone grafting is the gold standard, its ability to achieve bone union under relatively poor conditions in the recipient site such as large bone defects or atrophic non-union is not promising. An autologous bone graft contains cells including bone marrow mesenchymal stem cells (MSCs) and several growth factors which induce bone formation in a native bone structure. This raises the possibility that augmentation of these factors could enhance the ability of a graft to achieve bone union even under poor conditions.

Members of the fibroblast growth factor (FGF) family, which comprises 23 subtypes, are present in most tissues throughout the body and exert a variety of physiological effects, while their abnormal expression causes human genetic diseases such as achondroplasia or thanatophoric dysplasia [1]. Among the FGF family members, FGF-2 is known as a potent angiogenesis inducer and also has bone-forming capacity. Several animal studies have demonstrated its ability to enhance bone union. Kawaguchi et al*.* reported that a single local application of FGF-2 promoted bone healing in the femur of normal and diabetic rats [2]. In addition, FGF-2 stimulated healing of segmental bone defects in rabbits [3] and accelerated fracture healing by enhancing callus remodeling in experimental dog tibial fractures [4]. Moreover, human clinical trials have been conducted to test whether bone union can be accelerated by local injection of recombinant human (rh)FGF at the fracture site [5].

Among the cells in the bone, MSCs have the ability to differentiate into multiple lineages and are relatively easy to obtain although it is necessary to culture the primary cells to obtain sufficient numbers for clinical applications. MSCs have the capacity to differentiate into osteoblasts and therefore have become one of the most promising cell sources in bone regenerative medicine. In critical-sized segmental defects in the femurs of adult athymic rats, there was significantly more new bone formation at 12 weeks, and the bone formed was stronger, in defects treated with mesenchymal stem cell-loaded ceramic scaffolds than in scaffolds without MSCs [6]. Furthermore, MSCs have the possibility to transmit or release various conductive factors to induce new vessel and bone formation [7–9].

We hypothesized that the combination of autologous bone grafts with FGF-2 or MSCs could achieve bone union even under quite poor conditions compared to conventional bone grafts. The purpose of this study was to verify whether bone union can be obtained using the combination of autologous bone grafting with FGF-2 or MSCs in an atrophic non-union model of a large bone defect in the rat.

## Methods

### Animals

Male Sprague-Dawley rats aged 12 weeks (*n* = 18, weighing between 310 and 340 g; Shimizu Laboratory Supplies, Kyoto, Japan) were used. They were housed with free access to food and water and allowed unrestricted weight bearing. This study was approved by the Ethics Committee for Experimental Animals of Hiroshima University. All animals were treated according to the guidelines stipulated by the Institutional Animal Care and Use Committee.

### Preparation of MSCs

A modification of Kotobuki’s culture method as described previously [10, 11] was used for the isolation and in vitro expansion of MSCs. The bone marrow was flushed out of the femoral and tibial marrow cavities of rats with a 21-gauge needle connected to a 10-mL syringe containing 10 mL of culture medium composed of high-glucose Dulbecco’s modified Eagle’s medium (DMEM; Life Technologies, Grand Island, NY, USA), 10 % heat-inactivated fetal bovine serum (FBS; Sigma-Aldrich Corp., St. Louis, MO, USA), and antibiotics (at a final concentration of 100 units/mL penicillin, 100 μg/mL streptomycin, and 0.25 μg/mL amphotericin B; Nacalai Tesque, Kyoto, Japan). The cells were incubated in a humidified atmosphere of 5 % CO_2_ and 95 % air at 37.0 °C. The medium remained unchanged for the first 7 days and was subsequently changed every 2–3 days. After 14–21 days, the cells had proliferated and reached confluence. The cells were then harvested using 0.25 % trypsin and 0.02 % EDTA, then rinsed twice with culture medium. To expand the MSCs, 2–3 × 10^5^ harvested cells were seeded into 100-mm culture dishes. On reaching confluence again, the cells were reseeded under the same conditions. We confirmed that these cells had multi-differentiation ability of osteogenesis, adipogenesis, and chondrogenesis [12].

### Surgery

**Fig. 1:**
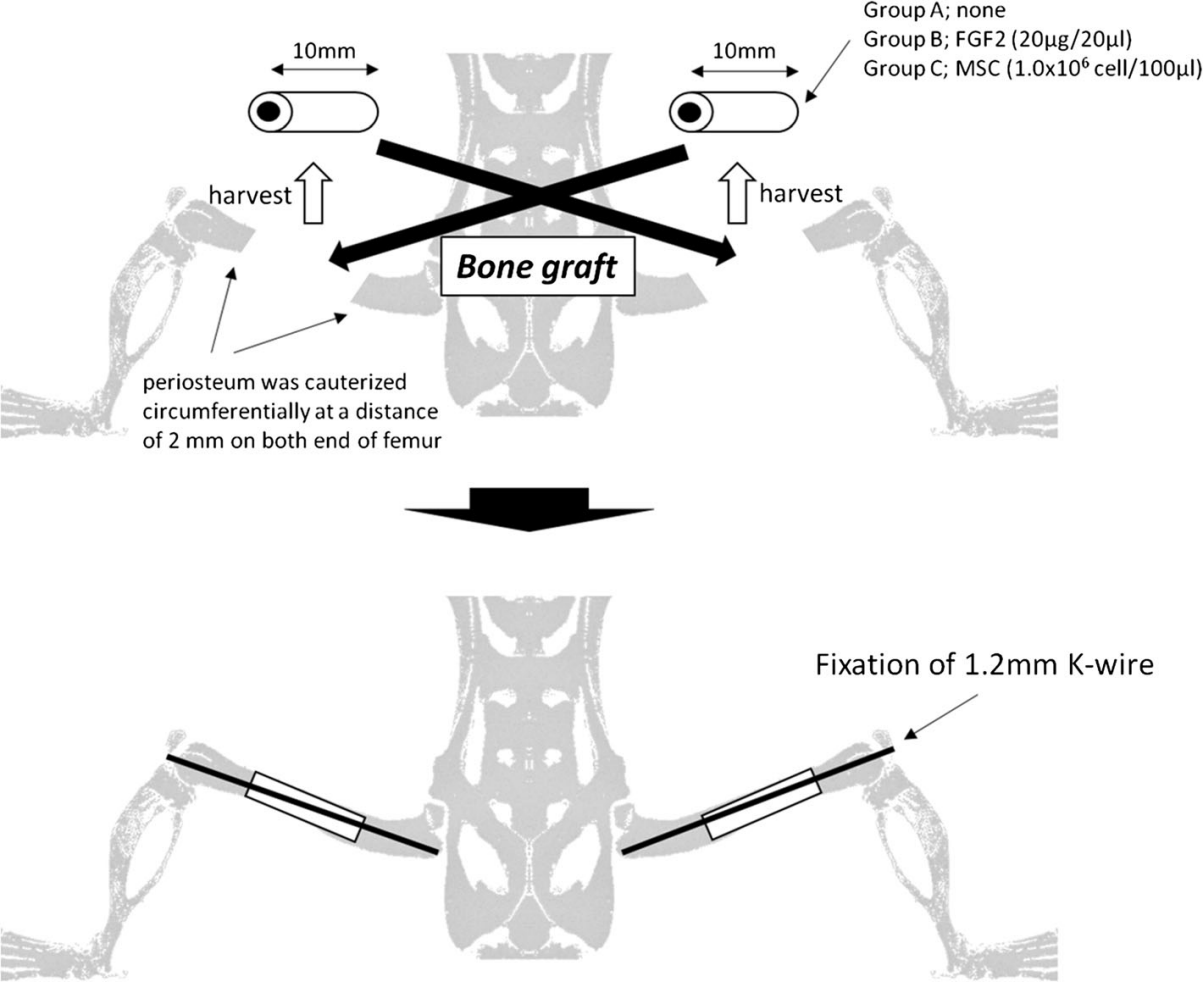
Atrophic non-union model of a large bone defect. In each rat, the bilateral femoral shafts were used for harvesting of the autologous bone graft. The mid-shaft of the femur was surgically removed and used as an autologous bone graft, 10 mm in length. The autologous bone removed from the right femur was transplanted to the left femur, and the left side was transplanted to the right side

All surgical procedures were performed under normal sterile conditions, and animals were anesthetized by intraperitoneal administration of sodium 5-ethyl-5-barbiturate (50 mg/kg). Non-union was induced in the bilateral femurs by creating a large bone defect and cauterizing the periosteum. Prior to surgery, the extremities were shaved and prepared in a sterile fashion. In each rat, both femurs were operated on and the femoral shafts on both sides were used for harvesting of the autologous bone for grafts. The bone grafts were harvested through a lateral incision. The muscle and periosteum were stripped circumferentially and the distal and proximal ends of the mid-shaft of the femur were osteotomized transversely using an oscillating power saw, then a 10-mm length of the mid-shaft of the femur was removed surgically. After removing the mid-shaft of the femur, the periostea were cauterized circumferentially at a distance of 2 mm on both ends of the femur to create non-union. An autologous graft of bone removed from the right femur was transplanted to the left femur, and the left side was transplanted to the right side. The bone grafts and femoral ends were then fixed in apposition with a single intramedullary 2.0-mm diameter K-wire, and the wound was closed in layers (Fig. 1).

The rats were randomized to three groups: group A (*N* = 6) received the autologous bone graft alone, group B (*N* = 6) was treated with an autologous bone graft together with FGF-2 (Peprotech, Rocky Hill, NJ, USA), and group C (*N* = 6) was treated with an autologous bone graft together with MSCs. The dose of FGF-2 was 20 μg per 20 μL, and MSCs were used at a density of 1.0 × 10^6^ cells/20 μL according to the previous reports [12, 13].

### Evaluation

Radiographic images were taken 2, 4, 6, 8, 10, and 12 weeks after surgery. According to the method described in a previous report [14], radiographic images were scored as follows: 0, no apparent hard callus; 1, slight intramembranous ossification; 2, hard callus without bridging of the fracture gap, fracture line is apparent; 3, hard callus with bridging of the fracture gap, fracture gap is noticeable; 4, unclear boundary between the newly-formed hard callus and existing cortical bone; and 5, remodeling. Radiographic images were scored on each set of four cortices, and all four cortex scores were summed.

The rats were sacrificed 12 weeks after surgery by intraperitoneal injection of a lethal dose of sodium pentobarbital, both femurs were harvested, and the K-wires were removed. The harvested bones were imaged by computed tomography (CT) using a SkyScan 1176 (SkyScan, Aarteselar, Belgium) with an isotropic voxel size of 18 μm, energy settings of 50 kV and 500 μA, and no filter. Then, the femurs were prepared for histology including toluidine blue staining. The femurs were fixed in 4 % paraformaldehyde for 24 h and then decalcified in KCX (FALMA, Tokyo, Japan) for approximately 12 h. After that, the femurs were dehydrated in alcohol and embedded in paraffin. The central section of each femur was cut into 5-μm-thick sections using a microtome, and the sections were stained with toluidine blue. According to the classification of Allen et al. [15], the degree of fracture healing was scored as follows: 0, pseudoarthrosis (most severe form of arrest in fracture repair); 1, incomplete cartilaginous union (retention of fibrous elements in the cartilaginous plate); 2, complete cartilaginous union (well-formed plate of hyaline cartilage uniting the fragments); 3, incomplete bony union (presence of a small amount of cartilage in the callus); and 4, complete bony union. Histological evaluation of fracture healing was scored on both the proximal and distal ends of the fracture.

### Statistical analysis

All results in this study are expressed as the mean ± standard deviation (SD). Comparison among three groups was performed using the Tukey–Kramer post hoc test. *P* values of less than 0.05 were considered to be statistically significant.

## Results

**Fig. 2:**
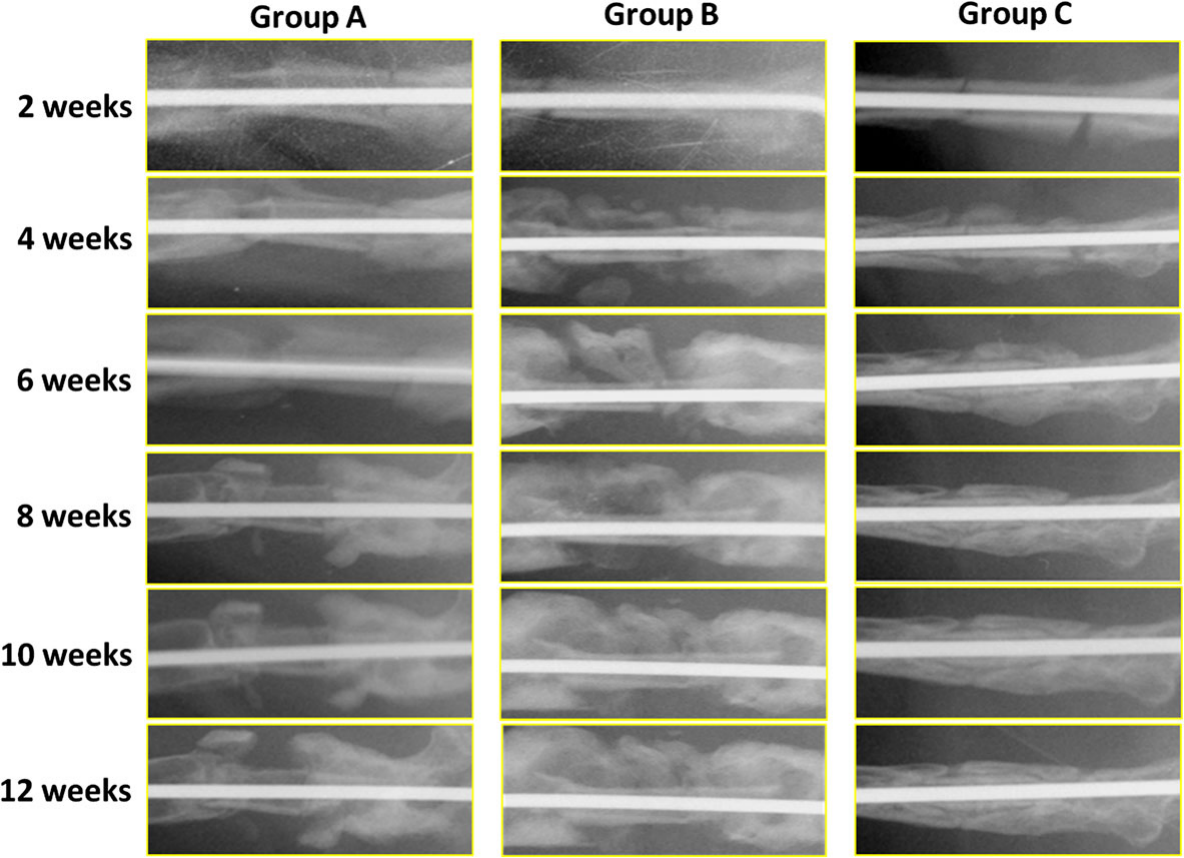
Plain radiogram in each group. Autologous bone grafting in a rat model was performed by transplanting the mid-section of the femoral shaft from the right femur to the left, and vice versa, with no further treatment (group A) or in conjunction with FGF-2 at 20 μg/20 μL (group B) or MSCs (1 × 10^6^ cells in 20 μL). In group A, there was good callus formation but no callus around the autologous bone graft. In group B, there was good callus formation but no bridging. In contrast in group C, there was good callus formation and bridging at 6 weeks, while by 12 weeks, the callus volume was reduced to less than that in groups A or B, but bone union was achieved at 12 weeks

**Fig. 3:**
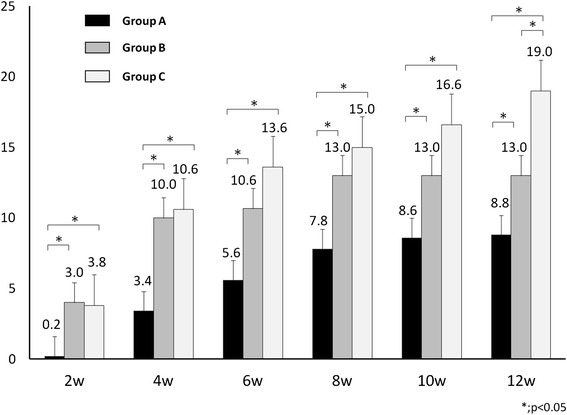
Radiographic scores in each group. The radiographic scores in groups B and C were significantly higher than that in group A and there was no significant difference between groups B and C at 2, 4, 6, 8, and 10 weeks. At 12 weeks, the radiographic score in group C was significantly higher than that in group B

**Fig. 4:**
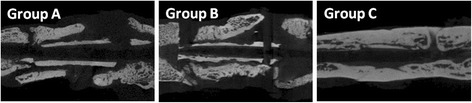
CT images in each group at 12 weeks. In CT images, there was abundant callus formation around the junction of femur and bone graft in all groups; however, there was no bridging callus formation in group A or B. In contrast, complete bone union could be observed in group C

In the radiographs at 2 weeks, no callus formation was evident in any group. However, there was obvious callus formation on the proximal and distal sides of the femur and on both sides of the autologous bone graft in groups B and C at 4 weeks while callus formation was only present on the proximal and distal sides of the femur with no callus on the autologous bone graft in group A. Bridging callus formation could be observed at 6 weeks in group C and was also observed at 10 weeks in group B. In group A, there was no bridging callus formation at 12 weeks. The bridging callus in group C was remodeled, and bone union was observed at 12 weeks (Fig. 2). The radiographic scores in groups B and C were significantly higher than that in group A and there was no significant difference between groups B and C at 2, 4, 6, 8, or 10 weeks. At 12 weeks, the radiographic score in group C was significantly higher than that in group B (Table 1) (Fig. 3). In CT images, there was abundant callus formation around the junction of femur and bone graft in all groups; however, there was no bridging callus formation in group A or B. In contrast, complete bone union could be observed in group C (Fig. 4).

**Table 1:** Radiographic and histological score

	Radiographic score						Histological score
	2 weeks	4 weeks	6 weeks	8 weeks	10 weeks	12 weeks	
Group A (*n* = 6)	0.20 (±0.45)	3.40 (±1.52)	5.60 (±2.30)	7.8 (±0.45)	8.60 (±1.95)	8.80 (±1.79)	8.60 (±2.07)
Group B (*n* = 6)	4.00 (±2.00) (a)	10.00 (±1.73) (a)	10.67 (±1.53) (a)	13.00 (±1.00) (a)	13.00 (±1.00) (a)	13.00 (±1.00) (a)	12.33 (±0.58) (b)
Group C (*n* = 6)	3.60 (±0.55) (a)	8.20 (±3.03) (a)	11.0 (±1.41) (a)	13.40 (±2.19) (a)	15.00 (±2.44) (a)	18.33 (±1.53) (a)(b)	18.40 (±1.14) (a)(b)

Radiographic score and histological score / mean (± SD) (*P* value)

Statistically significant differences were identified based on the Tukey–Kramer post hoc test (a, *P* < 0.05 compared to group A; b, *P* < 0.05 compared to group B)

**Fig. 5:**
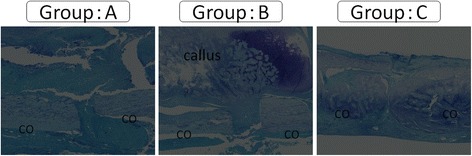
Histological analysis in each group at 12 weeks. In histological images, there was abundant callus formation in groups A and B, but any bridging at the fracture gap by the callus was not observed, and the fracture gap was still visible. In contrast, bone union was apparent in group C. Original magnification ×40

**Fig. 6:**
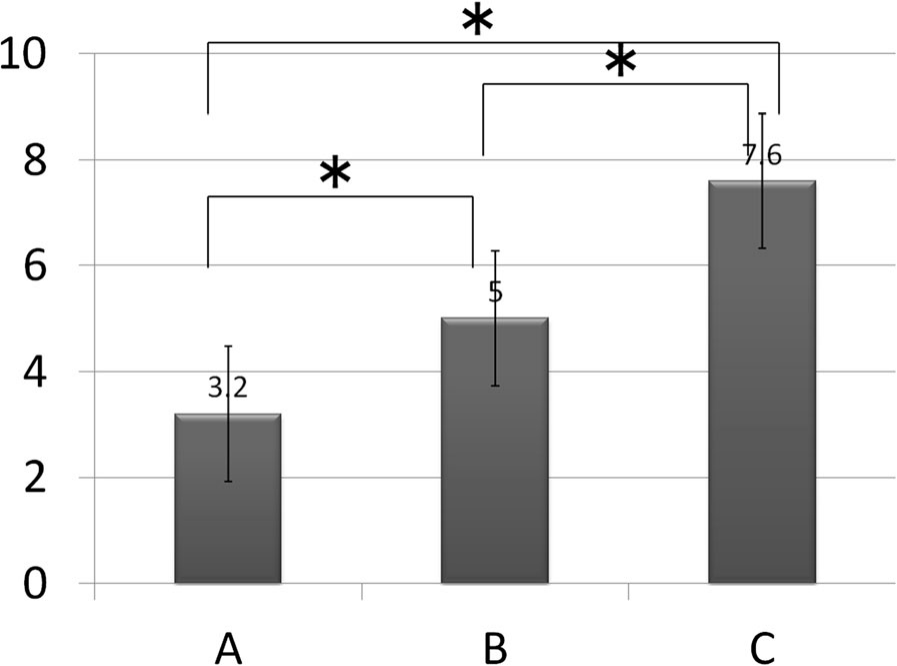
Histological score in each group at 12 weeks. The histological score was highest for group C in three groups. Group B was significantly higher than group A

The histological findings showed that in groups B and C, endochondral ossification with abundant chondrocytes and newly-formed woven bone were present around the junction of the femur and the bone graft. Bridging callus formation was observed in both groups B and C. However, the gap between the bone graft and the femur was not filled with callus in group B while bone union was observed in group C. In group A, the gap was filled with fibrous tissue (Fig. 5). The histological score in group C was significantly higher than all other groups, while the score in group B was significantly higher than that in group A (Table 1) (Fig. 6).

## Discussion

For the treatment of a bone defect or non-union, autologous bone grafting is recognized as the gold standard method, and in fact good results have been obtained in the clinical setting. However, autologous bone grafts may not necessarily give promising results in cases with large bone defects, poor conditions at the recipient site, or use of inappropriate fixation procedures between the fracture site and bone graft [16, 17]. In addition, harvesting of autologous bone for grafting has several disadvantages including limited harvest volume and pain or fracture at the donor site [18]. To overcome these problems, alternative procedures including the use of artificial bone or some modification of the bone grafting procedure such as the Masquelet technique have been developed [19]. However, a complicated procedure like the Masquelet technique requires multiple surgeries and the results are not promising. As for artificial bone, bone union is rarely obtained in large bone defects and consequently many trials to improve bone union using a combination of artificial bone and cells or growth factors have been conducted [20]. Ito et al. demonstrated that a composite of MSCs with an interconnected porous structure could enhance osteogenic potential, and that some injected MSCs were able to survive and differentiate into osteoblasts in the presence of an immunosuppressive agent, resulting in good bone formation [21]. A study by Nakasa et al. also demonstrated that a prefabricated vascularized bone graft created using FGF-2 administration and vascular bundle implantation into an interconnected porous structure composite had the ability to achieve bone union when transplanted into a bone defect, although further experimental studies are needed to enhance bone formation sufficiently for clinical use [22]. In addition, artificial bone itself has been improved. Indeed, these approaches to treatment at bone defect or non-union sites where it is difficult to obtain good results are important, but other ways to improve autologous bone grafting should also be explored. In this study, we evaluated the efficacy of the combination of an autologous bone graft in conjunction with MSCs or FGF-2 in achieving bone union under quite poor conditions.

Proliferation of immature mesenchymal cells is stimulated by FGF-2, but differentiation of osteoblastic cells is inhibited [23–25]. A study using injected ^125^I-labeled rhFGF-2 in hydrogel found that levels of rhFGF-2 were reduced to half at the fracture site within 1 to 2 days, with approximately 20, 3, and 1 %, respectively, of the ^125^I-labeled rhFGF-2 remaining at 1, 2, and 3 weeks at the local site [2, 26]. Therefore, the effect of FGF occurs at a relatively early stage of bone union during proliferation of immature mesenchymal cells. FGF promotes the induction and differentiation of bone morphogenetic proteins (BMPs), transforming growth factor β (TGF- β), and prostaglandins [2, 25, 27] so that FGF may have the ability to initiate the cellular and molecular cascade of the osteogenic process during fracture healing.

Recently, because of advances in tissue engineering techniques, cell-based treatments such as MSCs have begun to be used in the treatment of various diseases [28, 29]. MSCs have the capacity to differentiate into osteoblasts, and furthermore, MSCs also have the possibility to transmit or release various conductive factors which contribute to vessel and bone formation [30–32]. In bone union, MSCs play an important role in the bone regenerative mechanism. Granero-Molto et al. and Undale et al. demonstrated that transplanted MSCs enhanced callus volume, increased new bone volume, and improved biomechanical properties and could induce fracture healing by increasing biomechanical properties in mice and in a non-union nude rat model [33, 34].

In the current study, the use of an autologous bone graft in combination with MSCs resulted in good bone union while the combination with FGF-2 was not ultimately able to obtain bone union at 12 weeks although good callus formation was exhibited. The half-life of FGF-2 is reported to be in the range of 46 ± 21 min [35], and the physiological activity of FGF-2 is limited to the early phase after bone grafting. Therefore, in a situation which requires a long time to obtain bone union, the effect of FGF-2 might not last throughout the bone formation process.

In contrast, MSCs not only have the potential to themselves differentiate into osteoblasts but they also have the ability to induce differentiation of other progenitor cells and to recruit cells. Moreover, MSCs secrete humoral factors including cytokines and microvesicles. Throughout the process of bone formation, MSCs contribute to the differentiation into osteoblasts of both transplanted and endogenous MSCs and to the recruitment of these cells for bone formation by secretion of humoral factors, and these effects together subsequently result in complete bone union.

There are several criticisms of this study. Firstly, our non-union model in rat femur is quite different from the atrophic non-union model which has been previously reported [36]. We aimed to perform bone grafting into a poor condition site and evaluate the effect of MSCs or FGF-2 in combination with an autologous bone graft, using a non-union model in which the mid-shaft of the femur was surgically removed and the periostea were cauterized circumferentially at a distance of 2 mm on both ends of the femur. Typically, in clinical practice, an autologous bone graft is taken from the iliac crest. In the rat, the iliac bone is very thin, so that it is difficult to obtain an autologous bone graft from this site. Therefore, instead of the iliac bone, we used the harvested mid-shaft of the femur as an autologous bone graft, transplanting from the right femur to the left and from the left femur to the right. Under poor conditions for bone grafting, the combination of MSCs with an autologous bone graft resulted in good union. Secondly, in many previous studies, because the half-life of FGF-2 is very short, FGF-2 has been administered loaded into a gelatin sheet. In actual clinical applications, it is difficult to use gelatin sheets at the present moment and the administration technique is complicated. Consequently, in our study, a single-dose injection of FGF-2 or MSCs into the bone graft was performed in order to mimic the method of application that could be used in the clinic. In the clinical setting, the use of multiple applications of FGF-2 and MSCs is not feasible, and our results showed that a single-dose application of MSCs produced good bone union.

## Conclusions

In conclusion, the combination of MSCs with an autologous bone graft was able to produce good bone union for the treatment of a large bone defect or non-union.

## Abbreviations

FGF: Fibroblast growth factor
MSCs: Mesenchymal stromal cells
